# Expansion of KPC-3-producing *Klebsiella pneumoniae* ST15-KL19 after the COVID-19 pandemic in northern Portugal

**DOI:** 10.3389/fmicb.2026.1957369

**Published:** 2026-10-22

**Authors:** Ana Beatriz Gonçalves, Antónia Read, Isabel Neves, Natália Pinheiro, Valquíria Alves, Luísa Peixe, Ângela Novais

**Affiliations:** 1 UCIBIO, i4HB, Faculdade de Farmácia, Universidade do Porto, Porto, Portugal; 2 Clinical Microbiology Laboratory, Local Healthcare Unit, Matosinhos, Portugal; 3 Infection Control Unit, Local Healthcare Unit, Matosinhos, Portugal

**Keywords:** carbapenemase-producing *Klebsiella pneumoniae*, clonal expansion, Fourier-transform infrared spectroscopy (FT-IR), attenuated total teflectance (ATR), genomic surveillance, high-risk lineage, infection prevention and control, molecular epidemiology

## Abstract

**Introduction:**

Carbapenemase-producing (CP) *Klebsiella pneumoniae* are a major public health concern in Portugal, where the population has been dominated by KPC-3-producing ST147-KL64. Following preliminary detection of an unexpectedly high proportion of ST15-KL19 infections after the COVID-19 pandemic, we investigated the recent expansion and clinical relevance of this sublineage.

**Methods:**

All CP *K. pneumoniae* isolates recovered from hospitalized patients between 2022 and 2023 (*n* = 267) were characterized by antibiotic susceptibility testing and Fourier-transform infrared spectroscopy, *wzi* sequencing and multi-locus sequence typing. Representative ST15-KL19 isolates from a baseline (2019–2021) and the recent expansion (2022–2023) periods were selected for whole-genome sequencing and compared with publicly available global genomes to assess phylogenetic relatedness, temporal structure, and potential links to non-human reservoirs.

**Results:**

ST15-KL19 emerged as one of the most frequent CP *K. pneumoniae* sublineages, increasing from 23% to 35% and replacing ST147-KL64 over time. Genomic analyses identified two temporally distinct ST15-KL19 variants. Isolates from the recent expansion period (2022–2023) acquired *bla*_CTX–M–15_ and other plasmid-mediated aminoglycoside resistance determinants, concurrently with an IncR plasmid replicon. Phylogenomic analyses revealed that both variants were already circulating in the region before 2019. Together with the high colonization rates at admission (64%), our data suggest repeated introductions from external sources rather than in-hospital evolution. Global comparisons supported regional and intercontinental dissemination from a common ancestor, though a direct foodborne origin could not be established.

**Conclusion:**

This study documents the recent expansion of a previously minor CP *K. pneumoniae* sublineage, highlighting how shifts in healthcare ecology can favor adaptive variants or repeated introductions. Continuous molecular surveillance is essential to detect such emerging lineages early and to inform infection control and antimicrobial stewardship strategies.

## 1 Introduction

Carbapenemase-producing (CP) *Klebsiella pneumoniae* have been increasing throughout Europe over the last decade, with Portugal showing a particularly concerning rise in the rates of infections caused by carbapenem-resistant *K. pneumoniae* (European Centre for Disease Prevention and Control [ECDC], 2018, 2024b; Faria et al., 2024; Gonçalves et al., 2025a). Whole-genome sequencing (WGS) and phylogenomic analyses have clarified the role of specific sublineages within clonal groups (CG)15, CG258, or CG147 in driving the expansion of CP *K. pneumoniae*, underscoring the importance of monitoring clonal dynamics at local, regional, and global levels (Wyres et al., 2020). The COVID-19 pandemic in 2020 profoundly affected hospital dynamics, antimicrobial use, and infection prevention and control (IPC) practices, factors that have influenced the spread and evolution of CP *K. pneumoniae* in different settings (Du et al., 2025). The increase in infections caused by multidrug-resistant (MDR) *K. pneumoniae* and the strong selective pressure created by the increased use of carbapenems have potentially accelerated the spread of high-risk clones and facilitated the emergence and expansion of new resistant lineages (Abdelaziz Abdelmoneim et al., 2024; Golli et al., 2025).

Before 2019, the ST147-KL64 sublineage was the primary driver of *bla*_KPC–3_ dissemination in Portuguese hospitals (Rodrigues et al., 2016). This sublineage remained relevant in different hospitals over time (Spadar et al., 2022; Faria et al., 2024), although increasing clonal diversification has been reported, including ST13-KL3, ST348-KL62, ST231-KL51, ST15-KL24, and ST323-KL21 (Faria et al., 2024; Rodrigues et al., 2016; Spadar et al., 2022). Monitoring clonal dynamics over time is essential to understand transmission dynamics, relevant reservoirs, and the clinical impact of high-risk sublineages (Fox et al., 2024; Ikhimiukor et al., 2024). For this reason, we have been using Fourier-transform infrared spectroscopy (FT-IR) as a routine method for tracing MDR *K. pneumoniae* sublineages in real time in a hospital in northern Portugal over a 1-year period (Gonçalves et al., 2025b). In that study, we reported that 24% of the isolates causing infection in hospitalized patients belonged to ST15-KL19, placing it among the top three most frequent high-risk sublineages. However, this sublineage was relatively uncommon at both local (Spadar et al., 2022) and global levels (Rodrigues et al., 2023). In addition, it has been previously reported in poultry environments in our country (Mourão et al., 2023).

In the present study, we extended the epidemiological and genomic characterization of CP *K. pneumoniae* by focusing on ST15-KL19 isolates identified recently (2022–2023), aiming to investigate their expansion, transmission dynamics, clinical impact, and potential links to the food production chain.

## 2 Materials and methods

### 2.1 Ethical approval

This study was approved by the Local Commission for Information Protection and Security from the Local Healthcare Unit, Matosinhos, Portugal (Reference: 76/CLPSI/2022).

### 2.2 Bacterial strains identification and antimicrobial susceptibility testing

This study included 267 non-duplicate CP *K. pneumoniae* isolates recovered from 257 hospitalized patients at a district hospital in northern Portugal between 2022 and 2023. All isolates (*n* = 294) identified in that period were typed by FT-IR, as described (Gonçalves et al., 2025b), and only the first isolate of any given sublineage per patient within a 6-month period was included (Supplementary Table 1). Species identification and antimicrobial susceptibility testing were performed using automated methods, matrix-assisted laser desorption/ionization time-of-flight mass spectrometry (VITEK MS, bioMérieux, France) and VITEK 2 (CARD AST–N355, bioMérieux, France), respectively. Carbapenemase production was initially inferred by immunochromatographic assay (O.K.N.V.I. RESIST 5, Coris BioConcept, Belgium) and further confirmed by polymerase chain reaction (PCR) and Sanger sequencing (Rodrigues et al., 2016, 2014; Martins et al., 2025). Epidemiological and clinical data of predominant sublineages were collected, including age, sex, CP Enterobacteriaceae (CPE) colonization at admission, and in-hospital mortality (death occurring within the hospitalization period), either by all-cause (in-hospital mortality due to any cause) or infection associated (death resulting directly from the infectious process). Colonization was defined as the isolation of bacteria without clinical signs of disease, whereas infection required both microbiological isolation and clinical evidence of active disease.

### 2.3 *K. pneumoniae* sublineages identification

In this study, we analyzed non-duplicated *K. pneumoniae* isolates identified between January 2022 and December 2023. Capsular typing and sublineage identification were performed using our previously developed FT-IR machine-learning model (Gonçalves et al., 2025b).

A subset of these isolates (*n* = 127, collected from April 2022 to March 2023) was previously typed and extensively validated using *wzi* gene sequencing, multi-locus sequence typing (MLST), WGS, and pulsed-field gel electrophoresis (PFGE) during the model’s initial clinical implementation (Gonçalves et al., 2025b; Supplementary Table 1).

For the 140 additional isolates included in this extended study period, all FT-IR predictions were validated by *wzi* gene sequencing. Furthermore, MLST was performed on a subset of these additional isolates (*n* = 13, 1–2 representative isolates of either sporadic sublineages or newly introduced sublineages that subsequently caused outbreaks; Supplementary Table 1).

### 2.4 Characterization of ST15-KL19 isolates from northern portugal

In order to investigate the expansion of the ST15-KL19 sublineage, and track changes in phenotypic and genotypic profiles across time, extended antimicrobial susceptibility testing and WGS was performed for: (i) 11 isolates identified during the recent expansion period (five in 2022 and six in 2023) from patients hospitalized in different units and representing different months throughout each year; and (ii) five isolates previously identified in a baseline period (two in 2019, one in 2020, and two in 2021) to provide temporal context and assess whether earlier introductions preceded its recent expansion. All isolates caused infection in hospitalized patients.

Antimicrobial resistance phenotypes to 16 antibiotics were determined by disk diffusion according to the European Committee on Antimicrobial Susceptibility Testing [EUCAST] (2024) recommendations^1^. Interpretative extrapolations have been performed for tetracyclines. Tigecycline susceptibility results were interpreted using *Escherichia coli* clinical breakpoints as a proxy. Since EUCAST did not establish clinical breakpoints for tetracycline, susceptibility results for this antibiotic were interpreted according to Clinical & Laboratory Standards Institute (CLSI^2^). *E. coli* ATCC25922 was used as a standard strain for quality control.

Among the representative isolates selected for WGS, three genomes were retrieved from our previous work (Gonçalves et al., 2025b), while the remaining thirteen isolates were sequenced in the current study (Supplementary Table 1). For the newly sequenced isolates, genomic DNA was extracted using the Wizard Genomic DNA Purification Kit (Promega Corporation, USA) and sequenced on the Illumina NovaSeq 6000 platform (2 × 150 bp paired-end reads) at BMKGENE (Germany).

To ensure consistency, all raw datasets were processed through an identical downstream analytical pipeline (Supplementary Table 2). Raw reads were trimmed using Trim Galore v0.6.5dev and *de novo* assembled using SPAdes (v3.13.0 or v4.0.0). Genome assemblies were subsequently analyzed via Pathogenwatch v23.1.0^3^, where quality control was assessed using QUAST v5.0.2. Genomic characterization within the platform included the identification of antimicrobial resistance and virulence determinants via Kleborate v3.1.0, capsular (K-locus) and lipopolysaccharide (O-locus) typing using Kaptive v3.0.0b5, and plasmid replicon typing via PlasmidFinder v2.1. Finally, core genome MLST (cgMLST) profiling was conducted according to the platform’s integrated scheme.

### 2.5 Comparative phylogenomic analysis of ST15-KL19 in local and global contexts

Genomes from this study were compared with global genomes of the ST15-KL19 sublineage available in Pathogenwatch (*n* = 217, in June 2025) including isolates from different origin (human, poultry, and domestic animal). Whole genome comparisons were performed by generating a neighbor-joining tree based on the pairwise single nucleotide polymorphism (SNP) distances between genomes and a concatenated alignment of 1,972 core genes using Pathogenwatch (Argimón et al., 2021) and iTOL^4^ for tree representation.

### 2.6 Statistical analysis

Categorical variables, such as the proportions of specific high-risk clones (ST147-KL64 and ST15-KL19) across study periods, were expressed as absolute counts and percentages. Comparisons between proportions were performed using the Pearson’s Chi-square (χ^2^) test (or Fisher’s exact test when expected cell counts were < 5). Statistical significance was set at a two-tailed *p*-value < 0.05. All statistical analyses were conducted using Microsoft Excel formulas. The analysis unit was the individual non-duplicate bacterial isolate.

## 3 Results

### 3.1 Clonal diversity of CP *K. pneumoniae* in 2022–2023

The number of *K. pneumoniae* infections was similar in both years, *n* = 132 in 2022 and *n* = 135 in 2023 as well as the diversity of sublineages (*n* = 15–17), some of which caused outbreaks in 2022 (ST268-KL20, ST323-KL21) and 2023 (ST35-KL22KL37, ST198-KL10).

As previously reported (Gonçalves et al., 2025b), the most common *K. pneumoniae* sublineages were ST15-KL19, ST147-KL64, ST268-KL20, ST323-KL21 and ST198-KL10, which represented 82% of the total. Interestingly, ST147-KL64 and ST15-KL19 alone represented 54% of the whole sample (25% and 29%, respectively), but their relative proportions shifted significantly over time (χ^2^ test, *p* = 0.0333): ST147-KL64 decreased from 29% to 21%, while ST15-KL19 increased from 23% to 35%, indicating ongoing transmission rather than isolated events (Figure 1).

**FIGURE 1:**
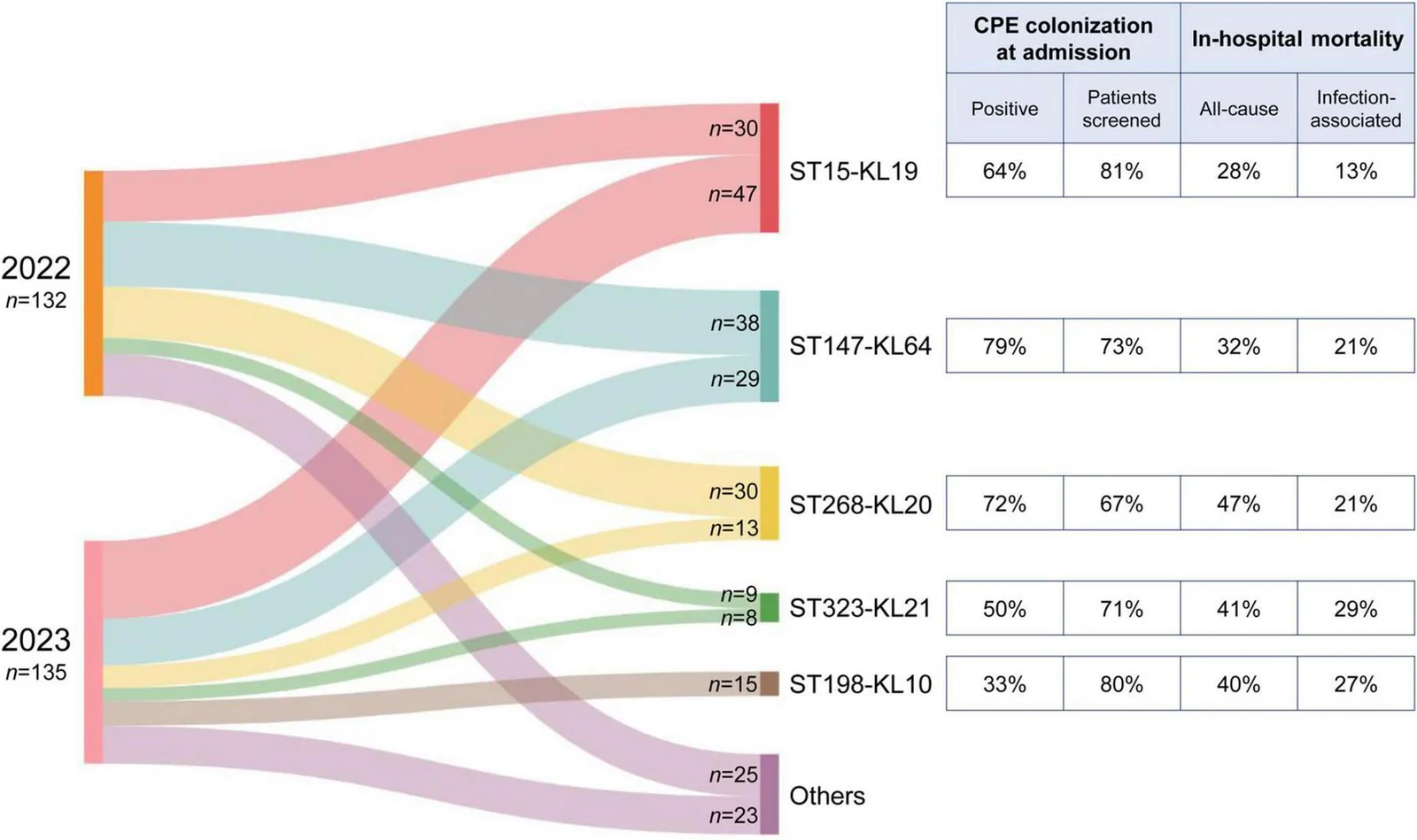
Frequency and diversity of sublineages of CP *K. pneumoniae* observed in a district hospital from northern Portugal (2022–2023). Made at SankeyMATIC.com.

Most CP *K. pneumoniae* isolates (96%; *n* = 257/267) were KPC-3 producers. Sporadic occurrences of other carbapenemases associated with ceftazidime-avibactam resistance were occasionally detected, including VIM-1 (*n* = 7; related to an outbreak by ST35-KL22KL37), IMP-22 (*n* = 1; ST29-KL30), and KPC-31 (*n* = 2; ST147-KL64) (Gonçalves et al., 2025a).

ST15-KL19 isolates exhibited consistent antimicrobial resistance profiles, being resistant to carbapenems (100% to ertapenem, 98.5% to imipenem and 97.1% to meropenem), third-generation cephalosporins (100% to cefotaxime and ceftriaxone), β-lactam/β-lactamase inhibitor combinations (100% to amoxicillin-clavulanic acid and piperacillin-tazobactam), fosfomycin (98.5%), ciprofloxacin (100%), and trimethoprim-sulphametoxazole (100%). All isolates (100%) of this sublineage were susceptible to ceftazidime-avibactam, amikacin and colistin.

### 3.2 Clinical and epidemiological data on predominant sublineages including ST15-KL19

Mortality varied across sublineages, with in-hospital infection-associated mortality ranging from 13.3% to 29.4% and all-cause mortality ranging from 28% to 46.5%. Marked differences in mortality were observed between sublineages, with ST323-KL21 showing the highest infection-associated mortality, while ST268-KL20 exhibited the highest all-cause mortality (Figure 1). Of the 211 patients with infections caused by one of these dominant sublineages, 159 (75%) were screened for CPE colonization at admission, of whom 106 (67%) were positive. The highest colonization rates were observed among patients infected with ST147-KL64, ST268-KL20, and ST15-KL19 (79%, 72%, and 64%, respectively). These major sublineages caused infections in adults across all age groups, particularly in the 60–79 (41%) and > 80 (42%) age groups.

### 3.3 Antimicrobial resistance patterns in ST15-KL19 isolates from northern Portugal

Antimicrobial susceptibility testing by disk diffusion performed on representative isolates (*n* = 16) confirmed resistance to third-generation cephalosporins (100% to ceftazidime and cefotaxime), carbapenems (100% to imipenem and ertapenem), piperacillin-tazobactam (100%), ciprofloxacin (100%) and trimethoprim-sulphametoxazole (100%), and consistent susceptibility to amikacin (100%) and ceftazidime-avibactam (100%). Most isolates were resistant to meropenem-vaborbactam and imipenem-relebactam (94%).

Resistance to cefiderocol, tetracyclines and aminoglycosides, however, was variable and temporally structured. Specifically, isolates from 2019–2020 were consistently resistant to cefiderocol (100%) and tetracycline (100%), and most to tigecycline (67%), while susceptible to gentamicin (100%) and tobramycin (100%). In contrast, isolates from 2021–2023 were all susceptible to tetracycline (100%), and mostly to tigecycline and cefiderocol (92%), but showed resistance to gentamicin (100%) and tobramycin (100%).

WGS analysis revealed that ST15-KL19 isolates belonged to the same sublineage (SL15) within CG15 and shared a common O1 antigen but differed in core genome sequence type (cgST), suggesting the existence of two ST15-KL19 variants, which are temporally structured (Supplementary Table 3). The isolates from 2019–2020 (cgST40521) carried the gene *tet(A)*, which correlated with resistance to tetracycline and tigecycline (Xu et al., 2021), alongside *qnrB1* and *bla*_OXA–9_. Plasmid replicon profiles included Col(pHAD28), IncFIA(pBK30683), and IncFII(pBK30683). In contrast, isolates from 2021–2023 (cgST6806) lacked *tet(A)* and *qnrB1* but acquired *bla*_CTX–M–15_, *bla*_OXA–1_, and the aminoglycoside resistance genes *aac(3)-IIa* and *aac(6’)-Ib-cr*, explaining their shift in aminoglycosides profile. These resistance genes were detected concurrently with an additional IncR plasmid replicon. Regarding virulence profiles, none of the sequenced ST15-KL19 genomes carried classical hypervirulence markers (such as *rmpA/rmpA2* or the *iuc* and *iro* loci). The only virulence determinant detected across both temporal variants was the yersiniabactin-encoding locus (*ybt 10*).

### 3.4 Phylogenomic analysis of global ST15-KL19 isolates

As of June 2025, 2226 ST15 genomes from 41 different KL-types were available in Pathogenwatch. Among these, ST15-KL112 (37%) and ST15-KL24 (29%) were the most frequent sublineages, while ST15-KL19 represented only 10%, which is in accordance with a global surveillance study (Rodrigues et al., 2023). ST15-KL19 genomes have been reported from 23 countries between 2007 and 2023, half of the isolates were collected in China (*n* = 106/217; 49%), which likely reflects a submission bias. Most (71%) of these were collected before 2020 and associated with humans (94%) with infections reported in 70% of cases.

Most ST15-KL19 public genomes carried carbapenemase genes (*n* = 168/217; 77%). Notably, CP isolates were predominantly from China (*n* = 97/106; 92%), all carrying *bla*_KPC–2_. In contrast, CP isolates from other countries showed a lower overall prevalence (*n* = 71/111; 64%), but a higher diversity of carbapenemase genes including *bla*_OXA–245_ (21%), *bla*_NDM–1_ (20%), *bla*_KPC–3_ (18%), *bla*_OXA–48_ (17%), *bla*_IMP–1_ (14%), *bla*_KPC–2_ (8%), and *bla*_VIM–1_ (1%). Additionally, most ST15-KL19 public genomes carried *bla*_CTX–M–15_ (*n* = 161/217; 74%) and genes conferring resistance to trimethoprim (78%), sulfonamides (76%) and tetracycline (60%).

SNP analysis confirmed the circulation of two temporally structured ST15-KL19 variants. Genomes within each period were highly related (0–9 SNPs between cgST40521 genomes from 2019 to 2020 and 5–11 SNPs among cgST6806 genomes from 2021 to 2023), whereas isolates from the two periods differed by 13–23 SNPs (Supplementary Table 4). Globally, genomes from this study were closely related with the public genomes (Figure 2, Supplementary Figure 1), with most (98%) differing from 3 to 97 SNPs (average = 53 SNPs). ST15-KL19 identified in this study were highly related (≤21 SNPs) to genomes carrying *bla*_KPC–3_ circulating in other hospitals from northern Portugal, France and the USA since at least 2017 (David et al., 2019). They differed 36–140 SNPs from those identified in China, supporting independent dissemination events. Notably, exact cgMLST matches for both our early (cgST40521) and recent (cgST6806) variants were already present among external genomes from northern Portugal dating back to 2018, whereas closely related international genomes (France, USA) displayed distinct cgSTs (Supplementary Table 3).

**FIGURE 2:**
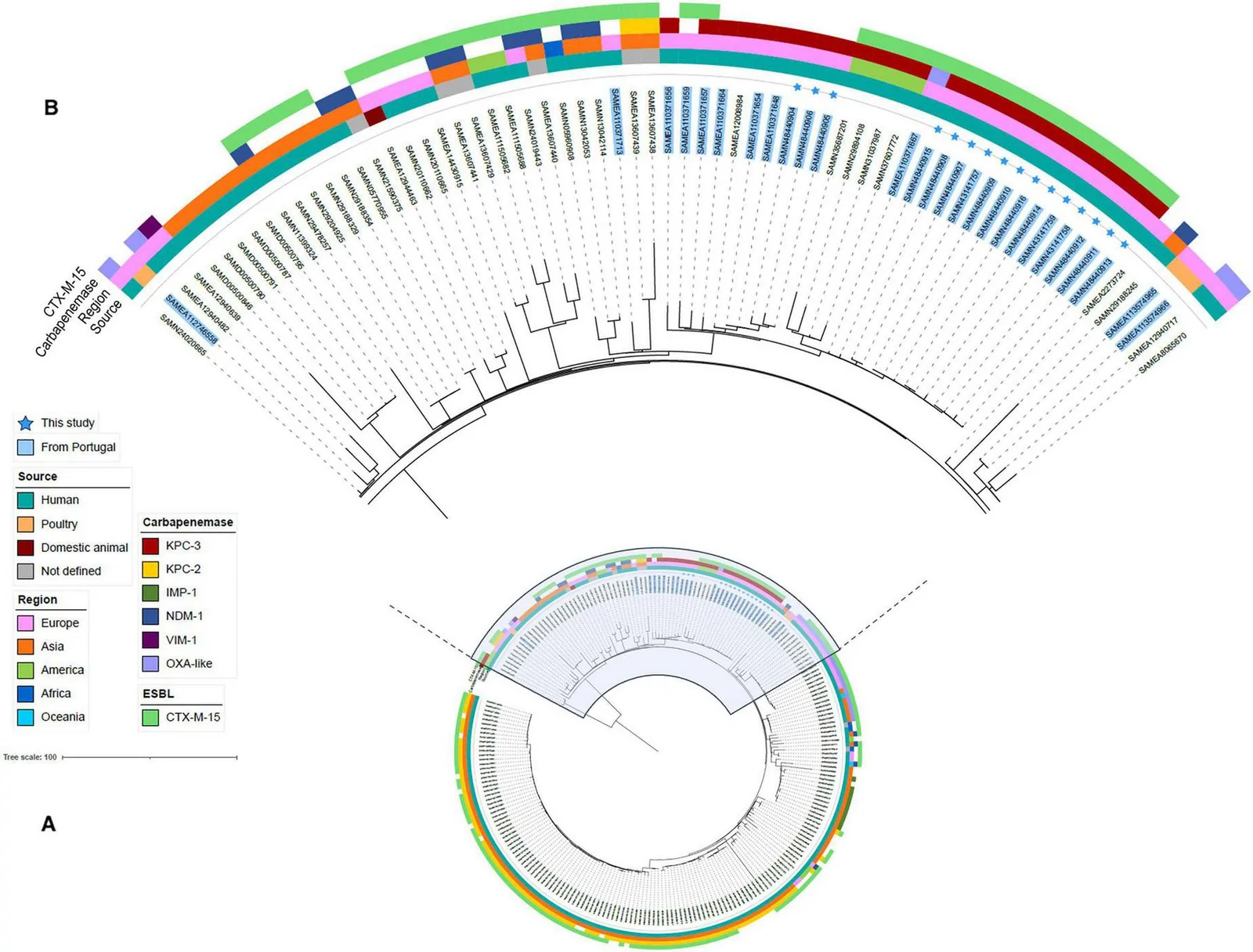
Core genome-based phylogenetic analysis of ST15-KL19 *Klebsiella pneumoniae* isolates. **(A)** High-resolution global neighbor-joining phylogenetic tree comprising all ST15-KL19 genomes from the Pathogenwatch database (*n* = 217). **(B)** Zoomed-in view of the focal clade highlighting the 16 study isolates alongside their closest public relatives from human infections (Portugal, France, USA) and poultry production in northern Portugal. Branch lengths represent pairwise SNP distances derived from the concatenated alignment of core genome sequences. Geographic regions and isolate sources are indicated. The full uncompressed phylogenetic tree is available as Supplementary Figure 1.

The genomes from this study differed in 35–49 SNPs from three public genomes from isolates identified in pre-slaughter chickens and poultry feces in Portuguese farms (2019–2022) (Mourão et al., 2024). These genomes did not carry carbapenemase genes but resistance determinants to aminoglycosides, fluoroquinolones, and tetracycline (Supplementary Table 3).

## 4 Discussion

This study reports a high number of infections by CP *K. pneumoniae* in a northern Portuguese hospital following the onset of the COVID-19 pandemic, accompanied by shifts in clonal composition and antimicrobial resistance patterns. Our data confirms the rapid expansion of the ST15-KL19 sublineage post-pandemic, contrasting with previous and contemporary local and global epidemiological data. These findings highlight dynamic clonal replacement and suggest that pandemic-related pressures may have contributed to the selection and dissemination of the ST15-KL19 sublineage.

We observed a high diversity of clones and the emergence of new sublineages (e.g., ST35-KL22KL37 and ST198-KL10) and carbapenemases (*bla*_IMP–22_, *bla*_KPC–3_ variants resistant to ceftazidime-avibactam) which complicates surveillance, outbreak detection, and the effectiveness of IPC measures, alongside treatment options and antimicrobial stewardship, thereby increasing the risk of intra- and inter-hospital dissemination (Allel et al., 2023; Gamaleldin et al., 2024; Ristori et al., 2024). Most of the sublineages detected have already been reported in previous studies in Portugal, some of which are contemporary (Spadar et al., 2022; Faria et al., 2024), and this increased epidemiological complexity is consistent with previous reports from other countries (Ficik et al., 2023; Abdelaziz Abdelmoneim et al., 2024; European Centre for Disease Prevention and Control [ECDC], 2024b; Golli et al., 2025), possibly reflecting the global impact of pandemic associated factors. Factors such as increased antimicrobial pressure due to widespread use of broad-spectrum and last-resort antibiotics, disruptions in IPC practices, high patient turnover and overcrowding in healthcare facilities, and ecological disturbances within hospital microbiomes might have favored sweeps and bottlenecks, promoting the survival and expansion of clones with enhanced fitness (Allel et al., 2023; European Centre for Disease Prevention and Control [ECDC], 2024a). Finally, the continuous dissemination of high-risk clones such as ST15-KL19 emphasizes the critical need for tight antimicrobial stewardship and the preservation of last-resort conventional antibiotics (such as newer β-lactam/β-lactamase inhibitor combinations and siderophore cephalosporins), ensuring their sustained efficacy (Mackow and van Duin, 2024).

The rapid expansion of ST15-KL19 may reflect either acquired adaptive advantages, including enhanced environmental survival and antimicrobial resistance, or repeated introductions of this lineage from external sources. Isolates from the expansion period (2022–2023) carried an additional IncR plasmid replicon concurrently detected with extended-spectrum β-lactamase and aminoglycoside resistance genes, which might have contributed to their selective advantage. In fact, most (74%) public genomes carry *bla*_CTX–M–15_ and acquisition of CTX-M-15-encoding plasmids has been determinant for the successful spread of other *K. pneumoniae* CG15 (Rodrigues et al., 2023), CG258 (Chen et al., 2014), and CG307 (Wyres et al., 2020) sublineages. ST15-KL19 infections were not associated with increased mortality compared to other sublineages and, in fact, we did not observe hypervirulence factors. However, high rates of colonization at admission suggest a potential community reservoir and repeated introductions from external sources that may have accelerated dissemination, paralleling observations reported in other regions (Cano et al., 2022; Fasciana et al., 2023).

ST15-KL19 has also been expanding in China, where it is the second most prevalent ST15 sublineage, rising from 2.1 to 5.8% in 2016–2019 to 10.9% in 2020 amongst all CP *K. pneumoniae* infection isolates (Zhao et al., 2022; Liu et al., 2022; Feng et al., 2023). In that region, this clone is considered high-risk due to the convergence of multidrug resistance and hypervirulence traits (*iucA*) associated with *in vitro* and *in vivo* virulence and worst clinical outcomes (Du et al., 2023). While genomes from this Chinese study were not available, the absence of virulence traits and the genetic distance from other publicly available genomes from China suggest independent emergence and transmission events.

Phylogenomic analyses confirmed that ST15-KL19 isolates from this study were closely related to genomes circulating elsewhere (northern Portugal, France, USA), indicating possible dissemination at a regional and intercontinental scale from a shared ancestral population (Schürch et al., 2018; David et al., 2019; Spadar et al., 2022). The pre-existence of the specific cgST6806 variant in the region prior to our recent hospital outbreak strongly suggests that the expanding clone did not emerge through local micro-evolution but represents an independent introduction and subsequent expansion of an already differentiated ST15-KL19 lineage. In line with this, our retrospective typing of a limited historical dataset from 2021 confirmed that this sublineage was already circulating in our setting (16% of available isolates).

Although closely related genomes were identified with poultry isolates from Portuguese farms, SNP-based analyses and phylogenetic distances did not support a direct genomic link to support a foodborne origin. However, given the limited number of public non-human genomes available at the time of this study (Mourão et al., 2023), these findings cannot conclusively rule out a potential food or animal reservoir. Broader One Health genomic surveillance with increased sampling of non-human reservoirs will be essential to accurately assess cross-environmental transmission routes. Besides, it is worth noting that conventional SNP thresholds for nosocomial transmission (Schürch et al., 2018; David et al., 2019) may be too stringent when investigating cross-environmental transmission, and broader criteria could reveal additional ecological links (Watt et al., 2025).

This study has some limitations that should be acknowledged. First, a relatively small fraction of the overall isolate collection underwent WGS, which was restricted by resource constraints. To mitigate potential sampling bias, sequenced isolates were carefully selected to be representative of the epidemiological diversity of the collection. Second, a robust long-term statistical temporal trend analysis across the pre-2022 period could not be performed due to shifts in surveillance sampling strategies and the limited representativeness of the 2019–2021 historical dataset available.

In summary, the recent expansion of ST15-KL19 suggests either an adaptive advantage of this sublineage and/or its continuous introduction from a common source, both of which have important implications for dissemination and IPC. Increasing clonal and resistance diversity poses critical challenges for surveillance, IPC, and therapeutic management. Implementing FT-IR in this setting proved to be an effective strategy to overcome the limitations of phenotype-based surveillance, allowing for real-time monitoring of lineage replacement and providing timely alerts to infection control teams regarding the circulation of emerging high-risk clones. Continuous molecular surveillance systems to detect and monitor high-risk sublineages in real time enable proactive interventions to limit transmission and maintain effective therapeutic and IPC strategies.

## Data Availability

The datasets presented in this study can be found in online repositories. The names of the repository/repositories and accession number(s) can be found in the article/Supplementary material.

## Footnotes

1
https://www.eucast.org/

2
https://clsi.org/

3
https://pathogen.watch/

4
https://itol.embl.de/
