# Supplementary material for: Expansion of KPC-3-producing Klebsiella pneumoniae ST15-KL19 after the COVID-19 pandemic in northern Portugal
Source: Front Microbiol. 2026 Oct 22;17:1957369. doi: 10.3389/fmicb.2026.1957369 (PMC13736621; doi:10.3389/fmicb.2026.1957369)
Supplement: Supplementary file 1 [file Image_1.pdf]

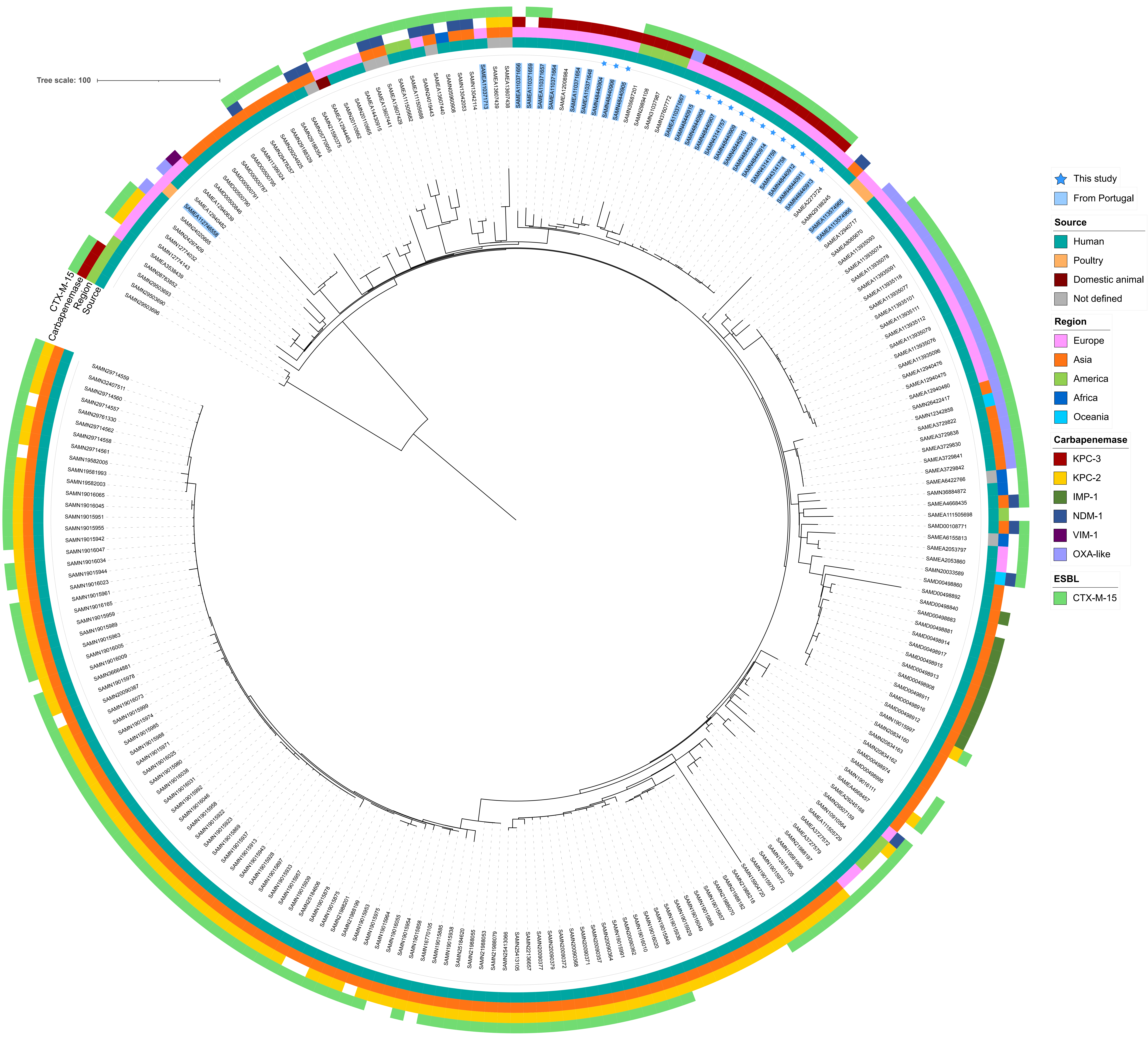

Tree scale: 100
CTX-M-15
Carbapenemase
Region
Source
This study
From Portugal
Source
Human
Poultry
Domestic animal
Not defined
Region
Europe
Asia
America
Africa
Oceania
Carbapenemase
KPC-3
KPC-2
IMP-1
NDM-1
VIM-1
OXA-like
ESBL
CTX-M-15

Supplementary Figure 1. Core genome-based phylogenetic tree of ST15-KL19 *Klebsiella pneumoniae* genomes.

Neighbor-joining phylogenetic tree based on the pairwise SNP distances of the concatenated alignment of 1,972 core genes between the 16 ST15-KL19 genomes from this study and all ST15-KL19 public genomes deposited in the Pathogenwatch database (*n* = 217).
